# Exploring burnout among preschool teachers in rural China: a job demands-resources model perspective

**DOI:** 10.3389/fpsyg.2023.1253774

**Published:** 2023-10-11

**Authors:** Na Zhao, Ming Huo, Wim Van Den Noortgate

**Affiliations:** ^1^China Institute of Rural Education Development, Northeast Normal University, Changchun, China; ^2^Faculty of Psychology and Educational Sciences, KU Leuven, Leuven, Belgium

**Keywords:** preschool teachers, job demands, job resources, job burnout, rural

## Abstract

Rural preschool teachers are increasingly experiencing job burnout, which could lead to their intention to leave and negatively impact education quality. This research explored the prevalence of job burnout among preschool teachers in rural China. It further investigated the potential influence of job-related characteristics on their levels of burnout. This study surveyed 10,581 preschool teachers across 34 counties in 18 provinces in China, utilizing multilevel structural equation models to analyze the situation and factors influencing job burnout. The findings indicate that the situation regarding job burnout among preschool teachers is not encouraging, particularly in the western areas and independent public kindergartens. Job resources were found to be associated with a reduction in burnout, while job demands had the opposite effect. The findings also revealed that job demands served as a mediating variable between job resources and job burnout. Moreover, the results also showed that reduced job burnout among preschool teachers was related to teacher cooperation, decision making, kindergarten resources and salary. On the other hand, role commitments, business issues, and classroom management were associated with increased burnout among preschool teachers. Furthermore, the impact of demands and resources on burnout was found to be intensified by kindergarten variables. To address the issue of burnout, it is essential to recognize the diversity and heterogeneity of kindergartens and take specific measures to reduce work demands while providing adequate and specific resources. Attention should be given to diversity and integration to ensure a positive work environment that can effectively prevent job burnout among preschool teachers.

## Introduction

The global importance of preschool education has led to efforts to ensure universal access to quality early childhood development, care, and pre-primary education by 2030 (UNESCO, 2019). Despite considerable efforts, quality concerns in early childhood education continue to endure (Duncan and Magnuson, 2013; Li et al., 2017). Early childhood education in rural areas in China still faces numerous challenges such as inadequate workforce, low social status of teachers, and job burnout (Hu et al., 2016; Liu, 2019).

Burnout, a significant issue in the workplace, is characterized by emotional exhaustion, depersonalization, and reduced personal accomplishment (Maslach and Jackson, 1981; Schaufeli et al., 2009). Job burnout is a common phenomenon among teachers. For example, more than 20% of teachers in Canada report experiencing burnout syndrome on a weekly basis (Fernet et al., 2012). Studies have shown that teacher burnout can result in decreased job satisfaction (Skaalvik and Skaalvik, 2010), reduced teaching self-efficacy (Roohani and Iravani, 2020), and increased turnover intention (Cephe, 2010). Moreover, Choi (2018) highlighted that burnout can also lead to both physical and mental health problems.

At present, preschool teachers are frequently involved in a broad range of tasks. These ranges are from planning courses beyond preschool education domains to preparing assessments and reports or fostering children’s abilities within the scope of early childhood education or beyond that (Bullough et al., 2014). However, these outstretched responsibilities are assigned under the context of low wages and benefits (Whitebook et al., 2016). Preschool teachers in rural China face a range of challenges that contribute to the risk of burnout syndrome (Gong et al., 2020). These challenges include an inhospitable environment, inadequate teaching materials, an excessive workload, a low social status, and inadequate pay (Yang, 2014). Job burnout has become increasingly severe among preschool teachers in China. A relevant study revealed that 53.2% of urban preschool teachers in China experience job burnout (Li et al., 2020). Such burnout can lead to reduced professional commitment (Buettner et al., 2016), and adversely affect teacher-child relationships (Ansari et al., 2022). Additionally, it is difficult for preschool teachers facing burnout to establish good family-school relationships (Fantuzzo et al., 2012). In China, there are few researches focusing on burnout among rural preschool teachers, particularly in studies that use large-scale data.

In research on job burnout, the Job Demands-Resources (JD-R) theory is a prevailing framework. The JD-R model classifies job characteristics into two categories: job demands and job resources (Demerouti et al., 2001). While previous studies within the JD-R model have explored factors related to teachers’ burnout (Bakker and Demerouti, 2007), research specifically addressing burnout among rural preschool teachers in China is sparse. Moreover, the mechanism through which specific job demands and resources impact their burnout remains unclear. Consequently, there is an urgent need for research that thoroughly investigates preschool teachers’ burnout in rural China.

In order to gain a more profound insight into the burnout problem and enhance the quality of the rural early childhood education workforce, this study employs comprehensive national data and advanced statistical methods, specifically the multilevel structural equation model (MSEM) (Preacher et al., 2010). Our aim is to present an overall view of preschool teachers’ burnout in rural China. Accordingly, given the focus on rural preschool teachers, we explore potential factors which could influence their burnout by applying the JD-R model, expecting to inform targeted interventions and, in turn, mitigate preschool teachers’ burnout.

## The JD-R model

This study utilizes the JD-R model (Demerouti et al., 2001) as a theoretical framework. This model posits that every occupation has unique job characteristics, which can be understood in multiple ways. The JD-R model emphasizes that the selection of specific variables has no strict limitation. This flexibility allows researchers to tailor indicators based on specific contexts or settings (Schaufeli and Taris, 2014).

### Job demands

Job demands include physical, psychological, social, and organizational requisites that require continuous physical or psychological effort or expertise and consequently entail particular physical or psychological expenses (Demerouti and Bakker, 2011). Podsakoff et al. (2007) divided job demands into two practical categories: hindrance and challenge demands. Hindrance demands impede work engagement and can lead to job burnout (Podsakoff et al., 2007; Dollard and Bakker, 2010; De Spiegelaere et al., 2014; Bakker and Demerouti, 2017), while challenge demands can foster individuals’ advancement, engagement, and innovative behavior (Rich et al., 2010; De Spiegelaere et al., 2015). This study primarily focuses on hindrance demands in the context of job burnout.

Studies using the JD-R model have examined the relationship between job demands and job burnout. Skaalvik and Skaalvik (2020) found a positive relationship between depersonalization and low student motivation, as well as conflicting values. Scanlan and Still (2019) studied emotional exhaustion in relation to job demands, such as emotional demands, shift work, and work-home interference. This research includes classroom management, administrative work, and role responsibilities as the indicators of job demands.

#### Classroom management

Classroom management involves the actions preschool teachers must take to handle disruptive student behavior and maintain order in the classroom. Kollerová et al. (2023) found that disruptive student behavior contributed to teacher exhaustion. Teachers reported significant challenges with discipline problems (Skaalvik and Skaalvik, 2017).

Dicke et al.’s (2014) study revealed a correlation between disruptive student behavior and higher levels of teacher job burnout. Huk et al. (2019) also found that student disrespect and inattention were associated with increased teacher job burnout.

#### Administrative work

Administrative work, including tasks such as paperwork, meeting minutes, and non-instructional training programs, has been the subject of various studies examining its relationship with burnout (Arvidsson et al., 2019). A survey in 2016 showed that primary teachers in England spent an average of 33.2 h per week on non-teaching tasks, leading to burnout from both administrative and teaching responsibilities (Higton et al., 2017). Another study investigated the number of administrative demands contributing to teacher burnout (Arvidsson et al., 2019), and a study found that administrative work demands significantly predicted teacher burnout (Lawrence et al., 2019).

#### Role responsibilities

Role responsibilities refer to the various tasks preschool teachers undertake in kindergartens, including teaching and childcare. Løvgren (2016) found that the clearness of job role could lower burnout. Carlotto and Câmara (2019) conducted a study with 679 Brazilian teachers and discovered that role responsibilities significant predicted burnout. Konukman et al. (2010) concluded that an excessive number of role responsibilities contributed to role overload, which is positively associated with job burnout.

### Job resources

The term “job resources,” as defined by Demerouti and Bakker (2011) defined “job resources” as the physical, psychological, social, and organizational aspects of a job that assist in: (a) successfully achieve work expectations and work objectives, (b) eliminate the negative impact of job demands on individuals, (c) build personal positive learning motivation and work attitude, and (d) develop personally on the long-term (Demerouti et al., 2001).

Kwon and Kim (2020) categorized job resources into three types: (a) organizational resources, (b) compensation resources, and (c) developmental resources, which are further examined in our study.

#### Organizational resources

Organizational resources refer to allocation, support, procedures, culture, and other elements provided by an organization. The Conservation of Resources (COR) theory posits that these resources are vital predictors of job burnout. According to the COR theory, abundant organizational resources enable employees to generate new resources, increase productivity, and prevent burnout (Hobfoll, 2001).

This paper identifies teacher cooperation, teacher-children relationships (professional resources), and kindergarten resources as examples of organizational resources. Teacher cooperation involves preschool teachers engaging in discussions, collaborations, and shared decision-making to address challenges. Studies have shown that teacher collaboration is associated with lower levels of burnout (Levine and Marcus, 2010; Skaalvik and Skaalvik, 2011; Dierking and Fox, 2012; Conley and Cooper, 2013; Al-Adwan and Al-Khayat, 2016).

The teacher-child relationship refers to the daily interaction between preschool teachers and children, including the nature of their communication, cooperation, and how children perceive their preschool teachers. Studies have shown that closer teacher-student relationships can help prevent emotional exhaustion (Simões and Calheiros, 2019). Additionally, high-quality teacher-student relationships can enhance teachers’ feelings of self-efficacy (Spilt et al., 2011), and improving these relationships can be mutually beneficial for students and teachers, potentially reducing burnout (Unterbrink et al., 2007; Rodríguez-Mantilla and Fernández-Díaz, 2017).

Kindergarten resources include equipment and related resources available to preschools. Kroupis et al. (2019) found that physical education teachers who were highly satisfied with their sports equipment reported lower levels of burnout. Similarly, Raikes et al. (2015) observed that insufficient kindergarten resources in certain regions were associated with lower levels of perceived personal accomplishment among teachers, leading to burnout. Van Tonder and Williams (2009) found that in South Africa, teachers were expected to handle heavy teaching loads without adequate resources, which contributed to burnout.

#### Compensation resources

Compensation resources refer to aspects such as earnings, which can serve as a source of motivation for work. Using the JD-R model, Bottiani et al. (2019) demonstrated that teachers in schools with lower salaries are more susceptible to burnout. Furthermore, Li et al. (2020) found a significant correlation between income satisfaction and burnout levels among Chinese preschool teachers.

#### Developmental resources

Developmental resources include decision-making processes and innovative instructional resources that have the potential to advance human development. In this study, instructional innovation and decision making are selected as developmental resources. Gorozidis and Papaioannou (2016) found that innovative instructional activities implemented by schools would promote a sense of achievement among teachers and prevent burnout syndrome. Evers et al. (2002) also suggested teachers who score low in instructional innovation activities experience high levels of emotional exhaustion and depersonalization, which can lead to feelings of inadequacy. Decision making, in this study, refers to the right to participate in the decision-making process of the kindergarten workplace. Previous research has found that the level of perceived decision making is negatively correlated with teacher burnout (Dworkin et al., 2003; Betoret and Artiga, 2010; Genc and Kalafat, 2016).

### The two processes of JD-R model

The JD-R model is based on two processes. The first process, known as the energy depletion process, posits that job demands can deplete individuals’ resources, leading to severe physical and mental health consequences (Bakker and Demerouti, 2007). This may ultimately cause individuals to experience job burnout syndrome. The second process, referred to as the motivational process, involves job resources that are essential for maintaining individuals’ mental health, as they enhance their work morale (Bakker et al., 2003) and increase job satisfaction and commitment (Bakker and Demerouti, 2014).

In regard to the characterization of job demands (Demerouti and Bakker, 2011), excessive job demands can lead to the continuous depletion of resources among preschool teachers, which can result in job burnout syndrome. Therefore, our study starts from the following hypothesis:

*H1*: Job demands are positively related to preschool teachers’ job burnout.

*H1a*: Classroom management is related to an increased preschool teachers’ job burnout.

*H1b*: Administrative work is related to an increased preschool teachers’ job burnout.

*H1c*: Role responsibilities are related to an increased preschool teachers’ job burnout.

According to the motivational process described in the JD-R model, the acquisition of ample job resources can prove beneficial not only for attaining work-related goals but also for fulfilling their psychological needs, ultimately reducing job burnout (Demerouti et al., 2001).

Thus, the second hypothesis was proposed:

*H2*: Job resources are negatively related to preschool teachers’ job burnout.

*H2a*: Teacher cooperation is related to a decreased preschool teachers’ job burnout.

*H2b*: Kindergarten resources are related to a decreased preschool teachers’ job burnout.

*H2c*: Teacher-children relationship is related to a decreased preschool teachers’ job burnout.

*H2d*: Salary is related to a decreased preschool teachers’ job burnout.

*H2e*: Instructional innovation is related to a decreased preschool teachers’ job burnout.

*H2f*: Decision making is related to a decreased preschool teachers’ job burnout.

**Figure 1:**
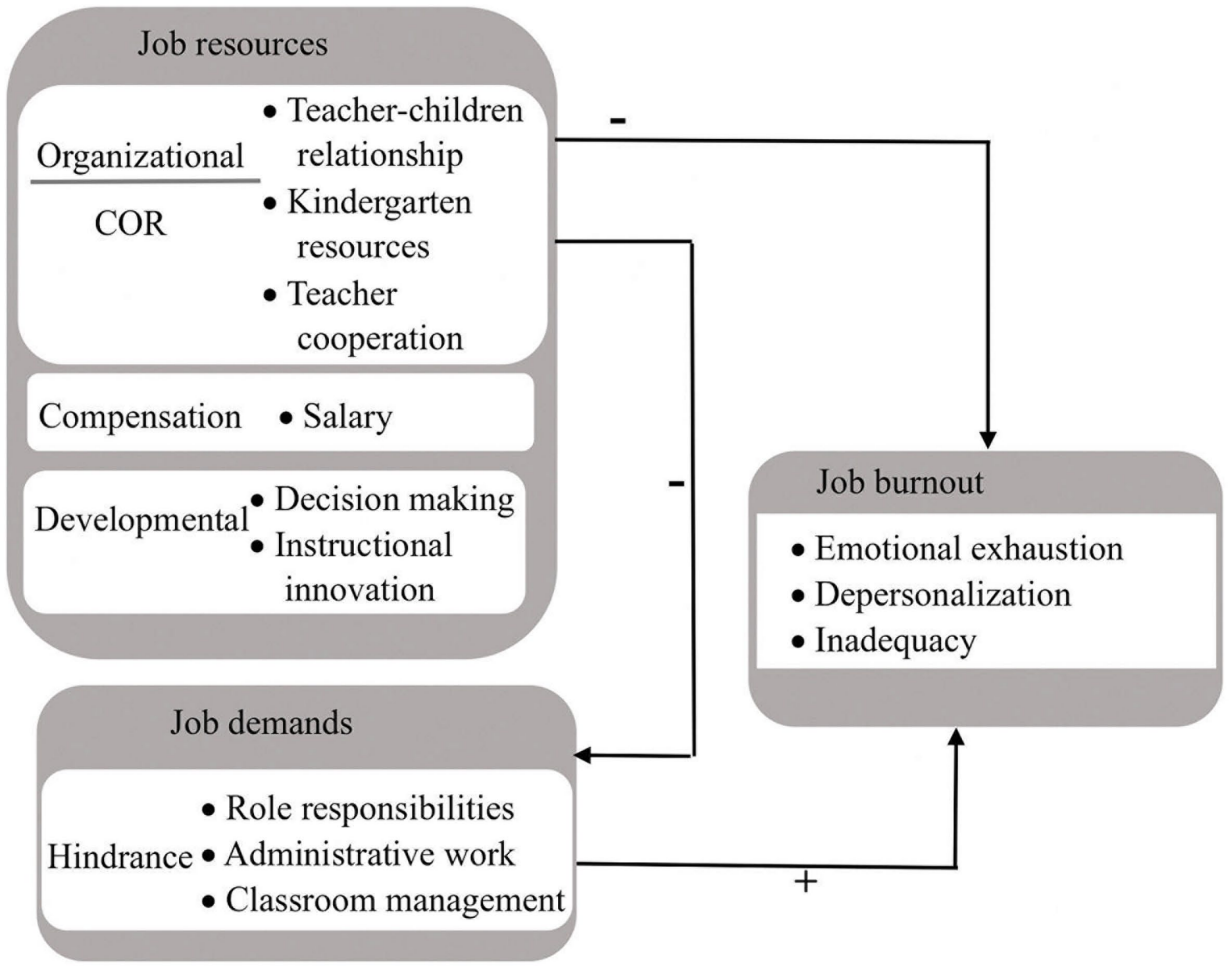
Conceptual model for job demands and resources to job burnout.

Previous research found evidence that job resources can potentially alleviate job burnout by effectively reducing the complexity of job demands (Bakker et al., 2005; Bakker and Demerouti, 2007). Consiglio et al. (2013) demonstrated that job demands played a mediating role in the relationship between self-efficacy and burnout, while Castanheira and Chambel (2010) established that job demands mediated the relationship between high-performance work systems and burnout. Therefore, we hypothesize the following in this study (see Figure 1):

*H3*: Job resources have a negative impact on job demands, with job demands serving as a mediating factor through which job resources can influence burnout.

## Method

Data were collected via online survey questionnaires. Quantitative data were addressed to gather the perception of burnout for kindergarten teachers and find the deeper mechanism of how selected predictors affected their burnout by employing MSEM.

### Data collection and participants

This study aimed to examine the labor and well-being conditions of teachers at the county level, with the participation of a total of 10,581 kindergarten teachers. Data collection took place between April and July in 2018, encompassing 1,201 kindergartens randomly selected from 34 counties across 18 provinces in eastern, central, and western China (refer to Table 1). We randomly selected half of the towns and villages in each county. Kindergartens were categorized based on their ownership as independent public kindergartens, private kindergartens and kindergartens attached to primary schools. The total number of kindergartens in each category was 385, 413, and 403, respectively.

**Table 1:** The details of questionnaires in each region.

Province	County	#Pop	GDP	#Kin	*N*	Province	County	#Pop	GDP	#Kin	*N*
Anhui	Jinzhai	52.90	1.43	48	1,522	Jiangxi	Dingnan	20.28	3.46	10	240
	Taihe	141.50	1.21	160			Xiushui	85.00	1.73	13	
Fujian	Changtai	22.31	9.46	9	372	Liaoning	Jianping	58.20	2.14	4	288
	Gutian	33.10	4.89	22			Liaoyang	57.06	3.50	46	
Gansu	Kang	17.92	1.15	22	381	Ningxia	Pengyang	19.45	2.07	18	539
	Lintao	51.48	1.25	69			Zhongning	34.15	3.74	14	
Guangdong	Haifeng	83.93	3.26	59	1,128	Shandong	Cao	167.5	2.40	41	1,368
	Wuhua	109.08	1.40	21			Guangrao	51.70	15.6	68	
Guangxi	Shangsi	21.18	2.72	3	60	Shanxi	Hongtong	75.67	2.19	98	686
	Zhaoping	35.44	1.16	2			Wuxiang	18.46	2.80	2	
Guizhou	Dafang	78.87	2.53	60	1,008	Shaanxi	Liquan	45.61	3.60	13	425
	Xingren	41.85	3.17	20			Yang	38.70	2.75	35	
Henan	Dancheng	99.53	2.43	14	701	Sichuan	Gulin	70.06	2.00	43	729
	Qinyang	43.78	8.68	58			Wusheng	59.12	3.44	46	
Hunan	Chenxi	46.30	2.35	17	428	Yunnan	Lufeng	42.70	3.00	5	177
	Hengshan	38.93	3.85	48			Luxi	44.05	2.01	3	
Hubei	Dawu	62.32	1.92	12	154	Chongqing	Fengjie	91.28	2.98	98	375

#Pop, #Kin and N, respectively, stands for the population size for each county (the measurement unit is ten thousand), the number of participating kindergartens and the number of questionnaires in each province (across counties). GDP refers to the Gross Domestic Product per capita (in ten thousand yuan).

We sent invitations to all full-time preschool teachers, allowing them to voluntarily complete questionnaires online. Data collection was made online via wenjuanxing, a professional on-line survey platform^1^. Following this, we conducted the data cleaning process, with the objective of screening careless or insufficient effort responses.

Of the participants, 97.34% were female, with ages ranging from 20 to 50 years (96%) to over 50 years (4%). Approximately 40.57% of participants graduated from normal schools, and 6.48% obtained a bachelor degree. Additionally, around 37.44% of the sample had less than 3 years of teaching experience, while 30.39% had between 4 and 10 years of teaching experience. The Academic Review Board of the Faculty of Education at Northeast Normal University, committed to upholding research ethics, approved the study.

#### Measures

##### Job demands

**Figure 2:**
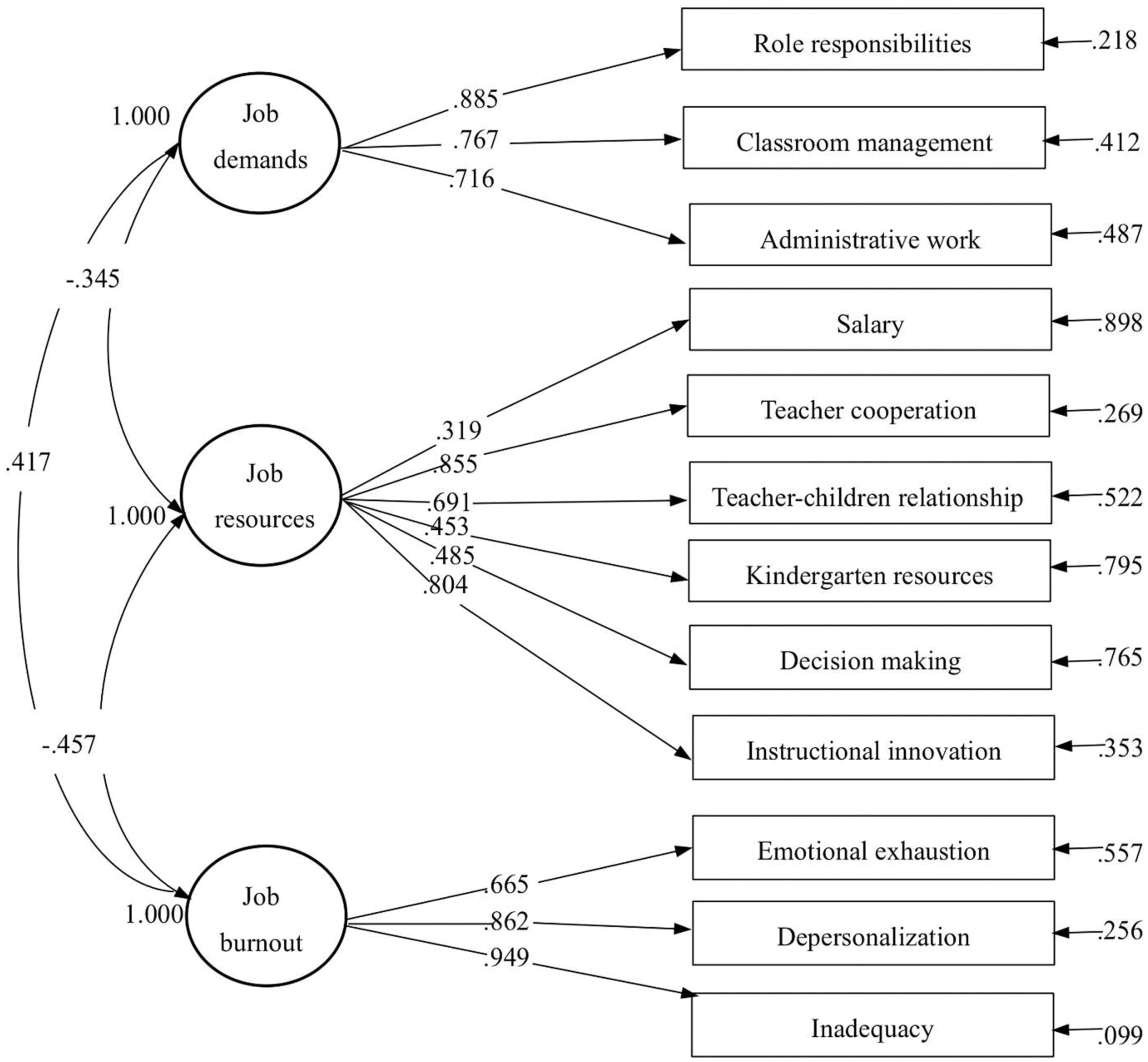
Result of measurement model.

To measure job demands, we used a scale proposed by Boyle et al. (1995). Each dimension was subdivided into nine levels, with corresponding values ranging from 1 to 9. All items can be seen in Figure 2. Items were classified into three subscales, and the average score of all items was used to represent the preschool teacher’s perceived job demands. Similarly, the score of each subscale was obtained by calculating the mean of all items within that subscale. A confirmatory factor analysis (CFA) was used via Mplus 7.4 and results were satisfactory (CFI = 0.911; TLI = 0.908; RMSEA = 0.047; SRMR = 0.072), and all factor loadings were appropriate, ranging from 0.641 to 0.920 (refer to the Data Analysis section for additional information on goodness-of-fit indices).

##### Job resources

The measurement of job resources employed the scale introduced by Johnson et al. (2007), whereby a 5-point Likert scale ranging from “strongly disagree” (1 point) to “strongly agree” (5 points) was utilized. Twenty items were selected, including salary (two items), instructional innovation (four items), teacher cooperation (six items), decision making (three items), kindergarten resources (two items), and teacher-children relationship (three items). Scores for job resources was determined by taking the average score of all responses. Likewise, the score for each subscale was derived by computing the mean of all items within that subscale. The CFA findings demonstrated a good fit with CFI = 0.921; TLI = 0.933; RMSEA = 0.063; SRMR = 0.054. Furthermore, the factor loadings were all satisfactory, ranging from 0.431 to 0.922.

##### Burnout

To measure burnout, the Bergen Burnout Inventory (BBI; Salmela-Aro et al., 2004) was utilized, where nine items were selected based on emotional exhaustion (three items), depersonalization (three items), and inadequacy dimensions (three items). Respondents rated the items on a 6-point scale from ‘strongly disagree’ (1 point) to ‘strongly agree’ (6 points). Similarly, scores for teachers’ burnout in each dimension were determined by calculating the mean of all items within each subscale. The three-factor model of burnout fit the data well through CFA: CFI = 0.916; TLI = 0.902; RMSEA = 0.045; SRMR = 0.055. All factor loadings were also deemed reasonable, ranging from 0.665 to 0.949. Indicators of reliability and validity are calculated for each scale (Table 2).

**Table 2:** The reliability and validity in each scale.

	KMO	Cronbach’s alpha
Job Demands	0.895	0.901
Role responsibilities		0.753
Administrative work		0.652
Classroom management		0.904
Job Resources	0.893	0.885
Instructional innovation		0.767
Teacher-children relationship		0.907
Salary		0.892
Decision making		0.522
Teacher cooperation		0.783
Kindergarten resources		0.832
Job Burnout	0.914	0.896
Emotional exhaustion		0.745
Depersonalization		0.822
Inadequacy		0.787

#### Data analysis

Initially, the burnout data are explored using descriptive statistics, and subsequently, a structural equation model (SEM) is employed to examine the predictors associated with job burnout among pre-school teachers. The study first tests each measurement model before constructing the structural model of preschool teachers’ job burnout based on previous research hypotheses, using Mplus7.4 to analyze the data. The goodness of fit indices is given as follows: (a) TLI ranged from 0.9 to 1 (closer to 1 suggests a good fit), (b) CFI ranged from 0.9 to 1 (closer to 1 suggests a good fit), (c) RMSEA ranged from 0 to 0.08 (less than 0.05 suggests a good fit), and (d) SRMR ranged from 0 to 0.08 (less than 0.05 suggests a good fit).

##### MSEM analysis

The data we have gathered has a multilevel structure, where teachers are nested in kindergartens, counties, provinces, and regions. Therefore, we also tried to provide a thorough understanding of the pathways that correlate with preschool teachers’ job burnout and related factors, by using MSEM.

To assess the extent to which observed variables varied between groups (at the kindergarten level), the intra-group correlation coefficient (*ICC*) was calculated initially, using the following formula:


$$ICC=\frac{{\hat{\tau }}_{00}}{{\hat{\tau }}_{00}+{\hat{\sigma }}^{2}}$$

*ICC* values ranging from 0.059 to 0.138 were considered to indicate a moderate correlation (Cohen, 1992), which necessitates the construction of a MSEM to ensure accurate data estimation in instances where the *ICC* value exceeds 0.059.

## Results

### The present state of job burnout among rural preschool teachers in China

**Figure 3:**
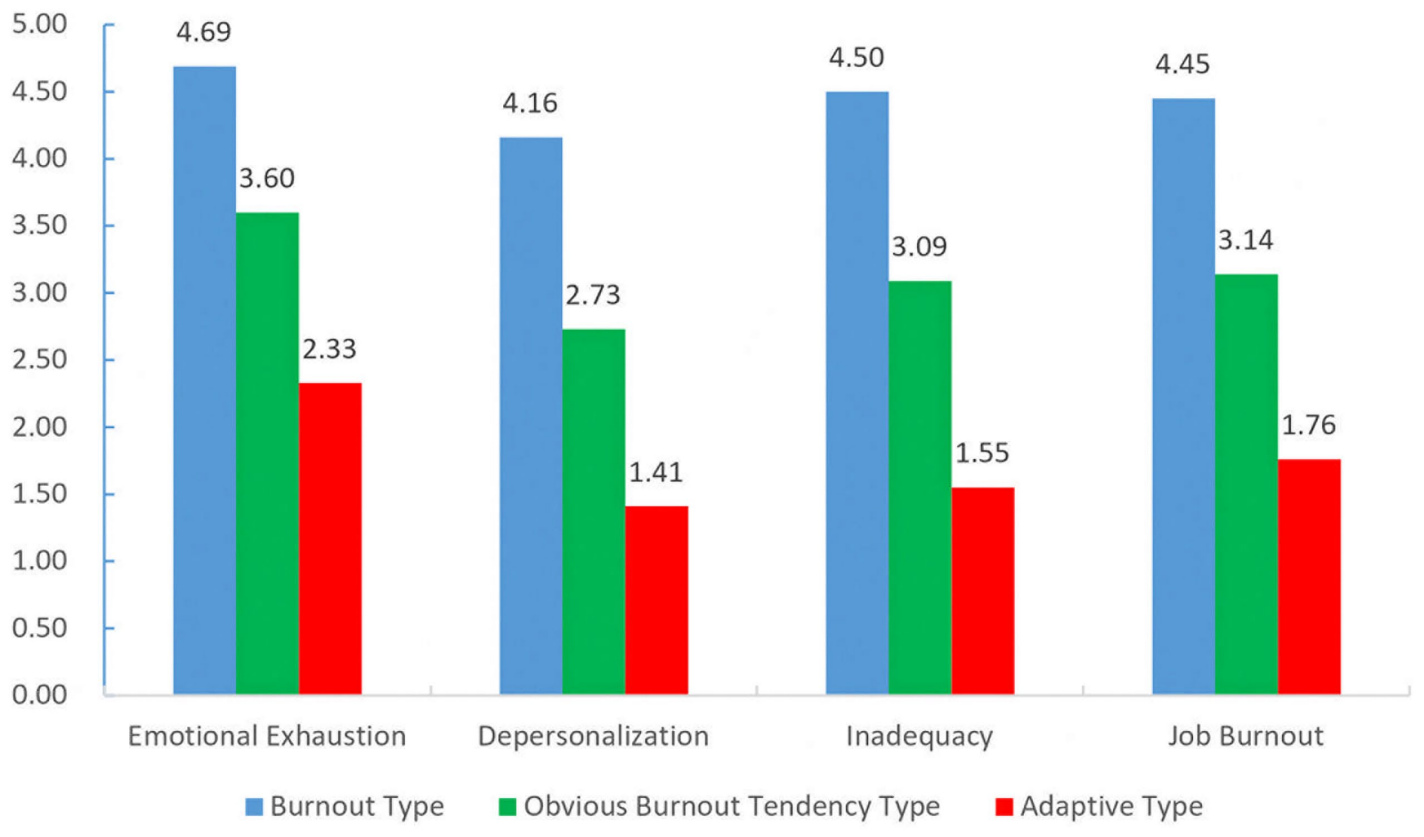
Mean value of every job burnout type.

Table 3 displays the highest perceived level of burnout was related to emotional exhaustion, while the lowest was associated with depersonalization. The questionnaire used in this study to assess preschool teachers’ burnout predominantly examined individual dimension scores. Based on these scores, cluster analysis via SPSS 22 categorized preschool teachers into three types: burnout type, obvious burnout tendency type, and adaptive type, with respective proportions of 26.9, 44.2, and 28.9%. This suggests that the proportion of burnout type and obvious burnout tendency type among rural preschool teachers was relatively high. Figure 3 illustrates that the burnout group exhibited high levels of emotional exhaustion, depersonalization, and inadequacy. This group may be viewed as demonstrating a lack of engagement, enthusiasm, and a passive work attitude, which needs to receive attention.

**Table 3:** Burnout among preschool teachers by geographical region.

Geographical region	M ± SD			
	Emotional exhaustion	Depersonalization	Inadequacy	Job burnout
Eastern (*N* = 3,156)	3.47 ± 1.21	2.64 ± 1.23	2.94 ± 1.29	3.02 ± 1.10
Central (*N* = 3,731)	3.41 ± 1.24	2.68 ± 1.24	2.92 ± 1.28	3.00 ± 1.12
Western (*N* = 3,694)	3.70 ± 1.20	2.86 ± 1.21	3.21 ± 1.24	3.25 ± 1.06
Total (*N* = 10,581)	3.53 ± 1.22	2.73 ± 1.23	3.02 ± 1.27	3.10 ± 1.10
*F* value	3.11***	3.47***	5.31***	4.45***

***p < 0.001.

### Regional disparities in factors associated with burnout

Table 3 presents the preschool teachers in the western region reported the highest degree of emotional exhaustion. Western preschool teachers also experienced the highest level of depersonalization and inadequacy. In contrast, central preschool instructors had the lowest sense of emotional exhaustion, regardless of their level of inadequacy or complete burnout.

As shown in one-way ANOVA, results indicate significant differences between preschool teachers from eastern, central, and western regions for each subscale as well as total burnout (all *p* < 0.001).

### Kindergarten property differences in correlates of burnout

Table 4 displays the variations in burnout levels among the participants based on the kindergarten’s ownership. According to the results, preschool teachers working in independent public kindergartens have reported the highest levels of burnout.

**Table 4:** Burnout among preschool teachers by kindergarten property.

Kindergarten Property	M ± SD			
	Emotional exhaustion	Depersonalization	Inadequacy	Job burnout
Independent public (*N* = 5,614)	3.71 ± 1.20	2.82 ± 1.23	3.16 ± 1.27	3.23 ± 1.08
Kindergarten attached to primary school (*N* = 1,594)	3.56 ± 1.20	2.69 ± 1.24	2.99 ± 1.26	3.08 ± 1.08
Private (*N* = 3,320)	3.20 ± 1.19	2.61 ± 1.23	2.81 ± 1.26	2.88 ± 1.11
*F* value	9.41***	10.21***	5.60***	4.24***

***p < 0.001.

As analyzed in one-way ANOVA, statistically significant differences were found in all subscales as well as in overall burnout, with *p*-values less than 0.001 for preschool instructors working in different kindergartens.

### Descriptive analysis of variables

Table 5 shows the mean of job demands, job resources, and job burnout, respectively. Preschool teachers in rural areas perceived a high level of role responsibility, with an average score of 5.85. The score for perceived work resources averages at 3.46, which is higher than the median of 3.00. Among all, teacher-child interaction is rated the highest, with an average score of 4.13. The average scores for innovative teaching practices, teacher collaboration, decision-making, and kindergarten resources were at 3.89, 3.75, 3.16, and 3.12, respectively.

**Table 5:** Descriptive estimation of variables.

	Representative item	Mean	SD
Job Demands		5.08	1.93
Role responsibilities	Safety responsibilities for children	5.85	2.08
Administrative work	Handling lots of administrative works	4.84	2.56
Classroom management	Disruptive student behavior	4.57	2.07
Job Resources		3.46	0.55
Instructional innovation	Teachers in our kindergarten are all greatly with the spirit of innovation.	3.89	0.70
Teacher-children relationship	Children behave well in our kindergarten.	4.13	0.74
Salary	I am satisfied with my income.	2.70	0.97
Decision making	I hardly have a say in kindergarten affairs.	3.16	0.77
Teacher cooperation	Teachers cooperate in teaching programme design.	3.75	0.70
Kindergarten resources	Inadequacy of equipment and resources.	3.12	1.04
Job Burnout		3.10	1.10
Emotional exhaustion	I often cannot sleep well because of work environment.	3.53	1.22
Depersonalization	I feel like I have less to give.	2.73	1.23
Inadequacy	My expectations for my work and performance have been lowered.	3.02	1.27

### Test of the measurement model for the predictors of job burnout

First, we analyze the fitting indices in the model, as a result (*CFI* is 0.902; *TLI* is 0.904; *RMSEA* is 0.061; *SRMR* is 0.063), suggesting a reasonable measurement model. Ultimately, the design of the measurement model is correct because standardized path coefficients are significant and the factor loading is in reasonable range (see Figure 2).

**Figure 4:**
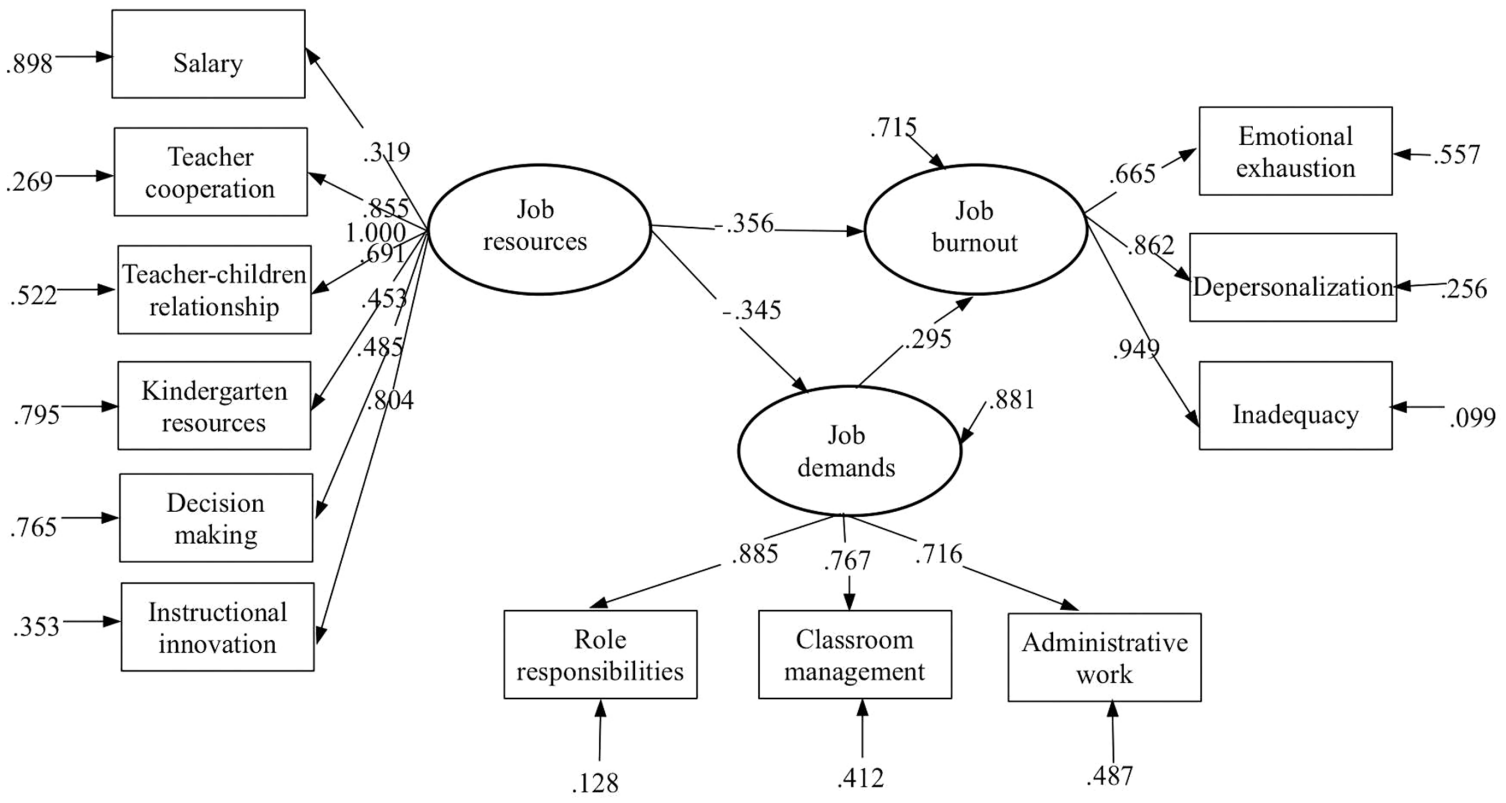
Result for job burnout model.

### Research hypothesis verification

The results reveal that (*CFI* is 0.918; *TLI* is 0.916; *RMSEA* is 0.072; *SRMR* is 0.076), suggesting reasonable structural model.

The structural model’s path coefficients reveal that job demands have a positive correlation with increased job burnout among preschool teachers, with a standardized path coefficient of 0.295 (as illustrated in Figure 4). On the other hand, job resources have a negative correlation with job burnout, with a standardized path coefficient of −0.356. As such, our hypotheses 1 and 2 are supported by the data. Additionally, Table 6 shows that reduced job burnout among preschool teachers is correlated with teacher cooperation, decision making, access to resources for kindergarten students, and salary. On the other hand, role commitments, business issues, and classroom management are associated with increased burnout among preschool teachers. However, it is clear that improvements in teaching methods and teacher-child relationships do not predict a decrease in job burnout among preschool teachers.

**Table 6:** Path coefficient of variables.

	Path coefficient	*P* value
Role responsibilities → Job burnout	0.060	*p* < 0.001
Classroom management → Job burnout	0.139	*p* < 0.001
Administrative work → Job burnout	0.093	*p* < 0.001
Salary → Job burnout	−0.073	*p* < 0.001
Teacher cooperation → Job burnout	−0.233	*p* < 0.001
Teacher-children relationship → Job burnout	0.145	*p* = 0.329
Kindergarten resources → Job burnout	−0.106	*p* < 0.001
Decision making → Job burnout	−0.118	*p* < 0.001
Instructional innovation → Job burnout	−0.014	*p* = 0.305

### Test of mediating effect

Baron and Kenny’s three-step test is used to test the mediating effect in this study (Baron and Kenny, 1986), and Bootstrap test also applies to ensure the correctness of the research conclusions.

As a result, the indirect effect value is −0.102 (a × b = −0.102) (*p* < 0.001), which suggests that job resources are correlated to a decreased job burnout by negatively influencing job demands, and job demands play a part of mediating effect in this process.

Additionally, the results of a Bootstrap test indicate that the mediating effect and direct effect are statistically significant at a significance level of 0.05. Hence, it can be concluded that job demands have a partial mediating effect between job resources and job burnout among preschool teachers. The mediating effect explains 22.3% of the total effect, implying that 22.3% of the impact job resources have on preschool teachers’ job burnout is attributable to the mediating effect (see Table 7). Therefore, null hypothesis H3 is supported.

**Table 7:** Mediating effect of job demands and its confidence interval.

	Standardized effect size	Percent	95% Confidence interval Bootstrap 2000 times
Job resources → Job burnout total effect	−0.457	–	[−0.478, -0.435]
Job Resources → Job burnout direct effect	−0.356	77.9%	[−0.378, -0.333]
Job resources → Job demands →Job burnout indirect effect	−0.102	22.3%	[−0.113, -0.092]

### Results of MSEM

The results showed by Mplus7.4 software report that the *ICC* values of the 12 observed variables are all greater than 0.059, indicating the importance of MSEM construction. Table 8 presents the fit indices suggesting that as compared to traditional SEM, the MSEM fits better.

**Table 8:** Fit indices in MSEM.

	*RMSEA*	*CFI*	*TLI*	*SRMR*
Traditional structural model of job burnout	0.072	0.918	0.916	0.076
Multilevel structural model of job burnout	0.056	0.922	0.911	0.070 (within-level) 0.062 (between-level)

#### Path analysis between latent and observed variables

Based on our data analysis, the within-group path coefficients for job resources or job demands and their observed variables are lower than those across groups. The between-group path coefficients of work burnout are, to varying degrees, enhanced in comparison to the within-group path coefficients. Consequently, there are noticeable differences in employment requirements, job resources, and burnout across kindergartens (see Table 9).

**Table 9:** *ICC* and path coefficients at the within- and between-group level.

	*ICC*	Within-level	Between-level	Within-level	Between-level	Within-level	Between-level
Role responsibilities	0.306	0.877	0.943				
Classroom management	0.162	0.739	0.904				
Administrative work	0.174	0.688	0.858				
Salary	0.271			0.263	0.606		
Teacher cooperation	0.201			0.807	0.965		
Teacher-children relationship	0.136			0.700	0.807		
Kindergarten resources	0.285			0.389	0.733		
Decision making	0.150			0.437	0.751		
Instructional innovation	0.189			0.800	0.943		
Emotional exhaustion	0.156					0.619	0.755
Depersonalization	0.113					0.841	0.921
Inadequacy	0.113					0.958	0.960

#### Path analysis between latent variables

The path coefficients for job demands and job burnout, job resources and job burnout, as well as job demands and job resources, are all higher at the between-group level. This suggests that job demands and job resources at the kindergarten level have a greater impact on job burnout than those at the level of preschool teachers. The substantial variation in job demands and job resources at the between-group level can be attributed to the aforementioned reasons. This indicates that while the samples selected between kindergartens are highly diverse in the two constructs, the samples chosen within kindergartens are relatively homogeneous in the two constructs of job demands and job resources (see Table 10).

**Table 10:** Path coefficients between latent variables in MSEM.

	Path coefficient (within-level)	*P* Value	Path coefficient (between-level)	*P* Value
Job Demands → Job burnout	0.200	*p* < 0.001	0.410	*p* < 0.001
Job Resources →Job burnout	−0.375	*p* < 0.001	−0.389	*p* < 0.001
Job Resources →Job demands	−0.294	*p* < 0.001	−0.671	*p* < 0.001

#### The proportion of variance explained

The variances of the residuals between groups for the observed variables are lower than those within groups. The ratio of explained variation at the within-group level and between-group level varies significantly for the 12 observed variables, particularly in areas such as kindergarten resources, decision making, salary, teacher cooperation, classroom management, and administrative works, where the proportion explained at the between-group level is notably higher (see Table 11). Thus, it can be concluded that observed variables like kindergarten resources exhibit notable differences among kindergartens.

**Table 11:** Results of residual and *R*^2^ in MSEM.

	Residual (within-level)	Residual (between-level)	*R*^2^ (within-level)	*R*^2^ (between-level)
Role responsibilities	0.231	0.111	0.769	0.889
Classroom management	0.453	0.183	0.547	0.817
Administrative work	0.527	0.264	0.473	0.736
Salary	0.931	0.633	0.069	0.367
Teacher cooperation	0.348	0.068	0.652	0.932
Teacher-children relationship	0.510	0.348	0.490	0.652
Kindergarten resources	0.849	0.463	0.151	0.537
Decision making	0.809	0.436	0.191	0.564
Instructional innovation	0.359	0.111	0.641	0.889
Emotional exhaustion	0.617	0.429	0.383	0.571
Depersonalization	0.292	0.152	0.708	0.848
Inadequacy	0.082	0.051 (*p* = 0.7)	0.918	0.930
Job demands	0.914	0.549	0.086	0.451
Job burnout	0.775	0.467	0.225	0.533

Also, the ratio of explained variation to job needs within groups is 8.6%, whereas at the between-group level, it is much higher at 45.1% and the case is the same with job burnout. As previously suggested, the inclusion of kindergarten-level variables significantly increases the proportion of job demands and job burnout that can be explained. This finding suggests that the variation associated with latent variables that cannot be fully explained at the within-group level can be accounted for by corresponding kindergarten factors at the between-group level.

## Discussion

Previous research often identified factors such as parents’ support, emotional needs, and professional development opportunities within the JD-R model as potential predictors of burnout syndrome (Demerouti and Bakker, 2011). In order to thoroughly explore the relationship between job characteristic variables and the burnout among rural preschool teachers, we have also introduced kindergartens’ climate as a predictor for discussion. Our examination of job demands variables include class management, role responsibilities, and administrative tasks. This study portrays a comprehensive view of burnout among preschool teachers in rural China and explored the underlying at both job demands and job resources levels that contribute to their burnout. The results indicate that certain job demands and resources variables may influence teachers’ burnout. These findings not only extend our understanding of burnout among rural preschool teachers, but also shed light on the future research in this area.

### Chinese rural preschool teachers’ burnout is not encouraging

This study conducts a comprehensive statistical analysis of the central concern of job burnout among rural preschool teachers. Findings indicate that job burnout is a significant problem among individuals with severe burnout problem and an obvious burnout tendency, requiring broad attention.

### Disparities in labor market factors and their correlation with burnout

Preschool teachers working in underprivileged areas or kindergartens with varying characteristics and locations are likely to experience more pressure conflicts. Consequently, Findings show that the regions where preschool teachers experience the highest levels of job burnout are predominantly in the western areas and autonomous public kindergartens where heavy workloads are common.

### Excessive job demands influence job burnout among rural preschool teachers

Based on the findings, the impact of job demands on preschool teachers’ burnout cannot be disregarded, as it not only affects the extent of burnout experienced but may also mediate the effect. According to the COR theory (Hobfoll, 2001), job demands correspond to the depletion of resources. As such, high and prolonged job demands will inevitably lead to the depletion of resources for preschool teachers, resulting in adverse effects on their physical and mental health, ultimately culminating in burnout.

Our findings highlight potential factors that policies can target to reduce teacher burnout and improve their well-being. For instance, the results indicate that preschool teachers who perceive classroom management as challenging are more likely to experience burnout. Consequently, classroom management has been associated with an increased job burnout, with the coefficients of 0.139. This aligns with previous research that increased teacher fatigue was significantly associated with disruptive student behavior (McCormick and Barnett, 2011; Bottiani et al., 2019). In addition, our findings suggest administrative work as a crucial factor influencing preschool teachers’ burnout. Moreover, preschool teachers rated high level of class management and administrative work demands, scoring 4.57 and 4.84, respectively. As a result, policies aimed at alleviating preschool teachers’ burnout and retaining their workforce would benefit from addressing these aforementioned job demands.

### Role demands obviously contribute to preschool teachers’ burnout

The primary function of a pre-school teacher is typically to provide instruction and education to their students. However, in remote rural areas with diverse and complex social and cultural backgrounds, their responsibility extends beyond that. As a result, they often take on excessive tasks and encounter ambiguous role assignments (Xu, 2017). Findings also reveal that preschool teachers undertake much more role responsibilities, which are important predictors of job burnout. With respect to findings in previous studies, Kilroy et al. (2016) identified that role conflict and role overload were significant predictors of emotional exhaustion and depersonalization. Our findings also show that they have the high perception of role demands, with 5.85 points. In short, the role responsibilities of preschool teachers in rural areas are overstretched, facing numerous role demands and unclear responsibilities, which can lead to work overload and job burnout.

### Teachers’ cooperation and decision-making abilities are the significant predictors of preschool teachers’ burnout

The relationship between job resources and reduced job burnout is evident, indicating that enhancing job resources in kindergartens is beneficial in alleviating the experience of job burnout among preschool teachers. Among the job resources, teacher cooperation and decision-making have the highest and second-highest associations with a decrease in job burnout, respectively (see Table 6). Unlike the k-12 education, where normally only one teacher manages a single classroom, preschool education often implements a team-teaching model due to the workload. However, preschool teachers seldom have enough opportunities for professional cooperative learning (Whitebook et al., 2014). This deficiency in collaborative skills may be associated with increased job burnout. Our findings suggest that in practical contexts, it is crucial to enhance teachers’ collaboration and facilitate more cooperative learning. Such measures might prove beneficial in reducing teacher burnout and increasing their overall well-being.

Regarding self-perceived decision-making, the average score is 3.16, which is relatively low. According to the motivational process (Bakker and Demerouti, 2007), the increase in job resources, such as decision-making, can effectively enhance work motivation. This, in turn, encourages individuals to be more engaged in their tasks and can help reduce their job-related fatigue.

### Kindergarten resources significantly influence preschool teachers’ burnout

The analysis reports that the mean of the availability of kindergarten resources is 3.12 points, requiring additional efforts to enrich kindergarten resources. When examining the influence on preschool teachers’ burnout, there is a clear negative relationship between kindergarten resources and burnout, with a coefficient value of −0.106. This result aligns with previous studies which suggested that a lack of teaching resources and challenging work conditions can exacerbate teacher burnout (Pithers and Fogarty, 1995; Kroupis et al., 2019). These results reaffirm that job resources, such as kindergarten resources, indeed correlate with reduced job burnout.

### Salary is not the primary predictor of preschool teachers’ burnout

Our results indicate that while salary does negatively influence preschool teachers’ burnout, its impact, with a coefficient value of −0.073, is smaller than other selected job resources factors. Borges et al. (2012) found that lower salaries were significantly related to the burnout among teachers. Our findings indicate that while salary does affect preschool teachers’ burnout, it is not the most decisive factor. Other human-centric elements, such as developing self-reflection skills through teacher collaboration and fulfilling self-esteem and self-actualization needs through decision-making process, are the more dominant predictors of preschool teachers’ burnout.

### Job demands serve as an important mediating variable

According to our report, job demands serve as a mediating variable. While job resources can motivate preschool teachers and enhance their sense of obligation and identity, high job demands without adequate job resources can result in a considerable energy drain and reduced work efficiency (Bakker et al., 2007). However, increasing the level of job resources alone may not fully resolve the problem of job burnout, as evidenced by the mediating role played by job demands. Therefore, there is a need to explore various approaches to address this issue, including improving the quality of job resources and keeping job expectations reasonable.

### The variables from kindergarten level should be considered as predictors affecting their burnout

The impact of various job characteristic variables on job burnout has been found to vary significantly, both within and between groups. The analysis of residuals shows that within-group residuals of each variable are higher than between-group residuals, indicating that the explanatory power of each latent variable for the observed variables is significantly enhanced after considering kindergarten variables.

Based on the path analysis, job demands and job resources have varying effects on job burnout at both the within-group and between-group levels. The between-group path coefficient is higher than the within-group path coefficient, suggesting that job demands and job resources have a more significant impact on job burnout at the kindergarten level. Statistical results also reveal that preschool teachers in western regions experience higher levels of job burnout than their counterparts in the eastern and central regions. Additionally, there are differences in the levels of job burnout among preschool teachers in autonomous public kindergartens compared to other kindergartens. In conclusion, these differences in job burnout among kindergartens can offer useful insights for addressing this issue in the future.

## Conclusion

The findings of this study highlight a worrying level of job burnout among preschool teachers in rural China. Regardless of the regional or specific kindergarten characteristics, these preschool teachers are experiencing different level of burnout. Factors related to job demands, such as role commitments, business issues, and classroom management challenges, have been identified as key factors giving rise to the increase of burnout. However, factors regarding job resources, including teacher cooperation, self-perceived decision making, available kindergarten resources, and salary, are seen as potential alleviating factors, providing some relief from burnout.

In our research we tend to explore the mediating role that job demands played in the relationship between job resources and burnout, aiming to develop more effective interventions to solve burnout problems. Additionally, we sought to clarify the influence of kindergarten-level factors on burnout mechanisms, using the JD-R model as the theoretical framework. Our findings suggest that tend to put emphasis on how kindergarten level factors influence the mechanism of burnout based on the JD-R model, denoting that the inclusion of kindergarten variables increases the impact of job demands or job resources on teacher burnout.

## Implications for policy and practice

The results of empirical analysis are different from the previous studies indicating that job resources are related to a decreased job burnout, while job demands have the opposite effect (Bermejo-Toro et al., 2016; Skaalvik and Skaalvik, 2020). However, our results realized from unique cultural background of rural areas in China, from special identity of preschool teachers in rural China, and from burnout issue urgently requiring to be solved in rural China are in great significance. It is pointed that job burnout is not only affected by job resources or job demands, but deeply affected by their interaction. Hence, we should carry out practical strategies and put emphasis on the process of mediating effect played by job demands to address job burnout problems. Additionally, we confirm that the inclusion of kindergarten variables changes the influence of potential factors on teacher burnout. Therefore, it is important to comprehend the differences in job burnout and working states of teachers at the kindergarten level, which are caused by variations in resource availability and workload across kindergartens. Regarding the conclusion, there are significant ramifications for enhancing preschool instructors’ mental health and lowering job fatigue. The indicators that government weighs when preparing for making strategies to respond preschool instructors’ burnout should be fully considered. It is probably not appropriate to raise single factor (i.e., wage) during the process of developing remedy methods to reduce their burnout. We should take into account the variations across kindergartens as well as job resources factors (i.e., teacher cooperation, decision-making, wage, etc) altogether as we proceed to thoroughly explore potential solutions to the burnout issue.

## Study limitations and future work

Despite providing a comprehensive and adequate national sample of preschool teachers, this study has limitations that should be acknowledged. First, this study only offers a snapshot of burnout among preschool teachers. Longitudinal research is urgently needed to understand how the phenomenon of burnout evolves over time in the population and to gain a better understanding of its progression within individual teachers. Furthermore, given the many studies on job burnout and the JD-R model, a systematic review of the literature could reveal deeper implications for both topics. However, conducting such a review was beyond the scope of this study. Future research should clarify how internal and external factors interact to influence preschool teachers’ job burnout. We should also consider how preschool teachers’ personal characteristics affect their job burnout in order to conduct a thorough and in-depth study of burnout syndrome.

## Data availability statement

The original contributions presented in the study are included in the article/supplementary materials, further inquiries can be directed to the corresponding author/s.

## Ethics statement

The studies involving humans were approved by the China Institute of Rural Education Development, Northeast Normal University, Changchun, China. The studies were conducted in accordance with the local legislation and institutional requirements. The ethics committee/institutional review board waived the requirement of written informed consent for participation from the participants or the participants’ legal guardians/next of kin because the Academic Review Board of the Faculty of Education at Northeast Normal University, committed to upholding research ethics, approved the study. Written informed consent was not obtained from the individual(s) for the publication of any potentially identifiable images or data included in this article because written informed consent for participation was not required for this study in accordance with the national legislation and the institutional requirements.

## Author contributions

NZ: Conceptualization, Visualization, Writing – original draft. MH: Methodology, Software, Supervision, Writing – review & editing. WV: Supervision, Writing – review & editing.

## Footnotes

^1^ https://www.wjx.cn/
